# Apolipoprotein E Homozygous ε4 Allele Status: A Deteriorating Effect on Visuospatial Working Memory and Global Brain Structure

**DOI:** 10.3389/fneur.2019.00552

**Published:** 2019-05-27

**Authors:** Janik Goltermann, Ronny Redlich, Katharina Dohm, Dario Zaremba, Jonathan Repple, Claas Kaehler, Dominik Grotegerd, Katharina Förster, Susanne Meinert, Verena Enneking, Emily Schlaghecken, Lara Fleischer, Tim Hahn, Harald Kugel, Andreas Jansen, Axel Krug, Katharina Brosch, Igor Nenadic, Simon Schmitt, Frederike Stein, Tina Meller, Dilara Yüksel, Elena Fischer, Marcella Rietschel, Stephanie H. Witt, Andreas J. Forstner, Markus M. Nöthen, Tilo Kircher, Anbupalam Thalamuthu, Bernhard T. Baune, Udo Dannlowski, Nils Opel

**Affiliations:** ^1^Department of Psychiatry, University of Münster, Münster, Germany; ^2^Department of Mathematics and Computer Science, University of Münster, Münster, Germany; ^3^Institute of Clinical Radiology, University of Münster, Münster, Germany; ^4^Department of Psychiatry, University of Marburg, Marburg, Germany; ^5^Core-Facility BrainImaging, Faculty of Medicine, University of Marburg, Marburg, Germany; ^6^Center for Mind, Brain and Behavior, University of Marburg, Marburg, Germany; ^7^Department of Genetic Epidemiology, Central Institute of Mental Health, University of Heidelberg, Mannheim, Germany; ^8^School of Medicine & University Hospital Bonn, Institute of Human Genetics, University of Bonn, Bonn, Germany; ^9^Centre for Human Genetics, University of Marburg, Marburg, Germany; ^10^Department of Biomedicine, University of Basel, Basel, Switzerland; ^11^Centre for Healthy Brain Ageing, School of Psychiatry, University of New South Wales Sydney, Sydney, NSW, Australia; ^12^Department of Psychiatry, Melbourne Medical School, The University of Melbourne, Melbourne, VIC, Australia; ^13^The Florey Institute of Neuroscience and Mental Health, The University of Melbourne, Parkville, VIC, Australia

**Keywords:** apolipoprotein E, visuospatial working memory, cognitive deficits, hippocampus, structural MRI, Alzheimer, major depression

## Abstract

**Theoretical background:** The Apolipoprotein E (APOE) ε4 genotype is known to be one of the strongest single-gene predictors for Alzheimer disease, which is characterized by widespread brain structural degeneration progressing along with cognitive impairment. The ε4 allele status has been associated with brain structural alterations and lower cognitive ability in non-demented subjects. However, it remains unclear to what extent the visuospatial cognitive domain is affected, from what age onward changes are detectable and if alterations may interact with cognitive deficits in major depressive disorder (MDD). The current work investigated the effect of APOE ε4 homozygosity on visuospatial working memory (vWM) capacity, and on hippocampal morphometry. Furthermore, potential moderating roles of age and MDD were assessed.

**Methods:** A sample of *n* = 31 homozygous ε4 carriers was contrasted with *n* = 31 non-ε4 carriers in a cross-sectional design. The sample consisted of non-demented, young to mid-age participants (mean age = 34.47; *SD* = 13.48; 51.6% female). Among them were *n* = 12 homozygous ε4 carriers and *n* = 12 non-ε4 carriers suffering from MDD (39%). VWM was assessed using the Corsi block-tapping task. Region of interest analyses of hippocampal gray matter density and volume were conducted using voxel-based morphometry (CAT12), and Freesurfer, respectively.

**Results:** Homozygous ε4 carriers showed significantly lower Corsi span capacity than non-ε4 carriers did, and Corsi span capacity was associated with higher gray matter density of the hippocampus. APOE group differences in hippocampal volume could be detected but were no longer present when controlling for total intracranial volume. Hippocampal gray matter density did not differ between APOE groups. We did not find any interaction effects of age and MDD diagnosis on hippocampal morphometry.

**Conclusion:** Our results point toward a negative association of homozygous ε4 allele status with vWM capacity already during mid-adulthood, which emerges independently of MDD diagnosis and age. APOE genotype seems to be associated with global brain structural rather than hippocampus specific alterations in young- to mid-age participants.

## Introduction

Alzheimer disease (AD) is a leading cause of dementia and associated with major cognitive and daily life impairments as well as psychiatric symptoms, such as depressed affect, agitation, and delusions (1). AD is a neurodegenerative disease that can be linked to extensive cerebral atrophy typically affecting structures involved in memory and cognition (2–4). Degenerations caused by AD are typically progressive, and mild cognitive impairment (MCI) can be found years before the outbreak of clinically relevant symptoms and constitutes a risk factor for AD (3). Both MCI and AD have been repeatedly associated with a deterioration of the hippocampus and the entorhinal cortex (4). Evidence from longitudinal studies suggests that these regions are the first to be affected by observable structural alterations in the course of AD (5, 6). Cerebral atrophies in these areas are accompanied by neurocognitive decline in several domains (7) as associative verbal memory (8), visuospatial memory (9, 10), and reasoning (11).

Brain structural changes associated with AD have also been shown to play a role in major depressive disorder (MDD) (12). Furthermore, a lifetime history of MDD poses a risk factor for developing AD later in life (13). However, the exact interplay between AD, MDD and brain structural alterations remains unclear.

The Apolipoprotein E (APOE) genotype is the strongest known single predictor of AD. APOE ε4 allele dose has been associated with higher risk and earlier onset of AD (14, 15). Heterozygous ε4 allele carriers are estimated to have a three-fold, and ε4 homozygotes an eight- to fifteen-fold increased risk to develop AD compared to subjects without an ε4 allele (14, 15). The risk effect of the APOE genotype has been replicated numerous times (16) and there is evidence that it is stronger in female compared to male (17).

The APOE genotype has further been associated with cognitive ability in elderly, non-demented samples. Evidence from studies contrasting healthy ε4 allele carriers with non-ε4 carriers suggests better performance in episodic memory (18, 19), working memory (20) and global cognitive functioning (21) in favor of non-ε4 carriers. However, empirical evidence is inconclusive with a considerable number of contradicting findings (21). Heterogeneity of findings may reflect methodological differences in ε4 allele status categorization (coding of zygosity), in the selection and operationalization of cognitive domains, as well as varying sample characteristics regarding age (21). Strongest effects of the APOE genotype have been found in elderly samples above 60 years and for APOE ε4 homozygotes (21). The role of the APOE status for the visuospatial domain has been discussed particularly controversially. There is some evidence for a visuospatial deficit in non-demented ε4 carriers that are mid-adult age (22) and elderly (23), however with no deficit found in a subgroup of age 80 and older (24). Although most studies investigating the APOE effect on cognition examine elderly samples deteriorating genotype effects on visuospatial performance can also be found in children (25) with some evidence for stronger deficits in girls (26). Contrasting findings exist (19) and a meta-analysis concluded that there is little evidence for group differences for the visuospatial domain between ε4 carriers and non-carriers (21). However, due to insufficient available sample sizes this meta-analysis did not carry out analyses comparing homozygous ε4 carriers with non-ε4 carriers, which might be an important biasing factor.

The effect of APOE status on cognitive ability appears to come along with functional and structural brain alterations. Evidence from an early PET study suggests inhibited glucose metabolism in ε4 homozygotes in temporal, frontal, and parietal regions, as well as the posterior cingulum already in a non-demented mid-elderly sample with normal cognitive functioning (27). Further, findings of hippocampal volume reductions are reported in healthy elderly ε4 homozygotes (28) as well as structural alterations in the temporal lobe in heterozygous ε4 carriers in a sample of healthy adults with a wide range of age (29).

A review (30) and a meta-analysis (31) further support these findings of structural alterations particularly in the hippocampal region in healthy elderly subjects as a function of the APOE genotype. However, evidence seems to be inconclusive regarding the role of heterozygous ε4 carriers as some studies find an ε4 dose effect, some find a similar degree and rate of cerebral atrophy for heterozygotes as for homozygotes while other studies find heterozygotes to be indifferent from non-ε4 carriers in respect to brain structural alterations (30). The degree to which studies differentiate the APOE genotype, and what genotypes are categorized as ε4 carriers vary. This has direct implications for methodological considerations of studies investigating APOE effects in that designs should differentiate ε4 allele dosage.

The evidence for a detrimental effect of the APOE ε4 allele on hippocampal structure as well as on vWM task performance raises the question if the hippocampal region might be the neuronal locus of the APOE genotype affecting the vWM. Although the visuospatial cognitive domain is commonly associated with the frontoparietal network (32) there are also studies linking it to the hippocampus. Performance in the Corsi block tapping task has been shown to be impaired in subjects with lesions in the right temporal lobe (33), and to be associated with augmented activation in the hippocampus in addition to frontoparietal regions (34). Other studies report that lower vWM capacity is associated with structural characteristics of the hippocampus, as lesions (35) and atrophy (36).

Brain structures and cognitive domains affected by the APOE genotype (as outlined above) are also closely related to detrimental processes involved in MDD (37, 38). There are studies linking MDD to a two-fold increased risk of AD (13) as well as at least one study suggesting that APOE genotype moderates this risk (39) and evidence linking APOE genotype to an increased risk for depressive symptoms (40). However, the exact interplay between the APOE gene, MDD, cognitive decline, and AD remain largely unresolved. Taylor et al. (41) investigated a sample containing MDD and healthy subjects and found no differences in various cognitive domains and structural MRI in function of ε4 presence (not differentiating between ε4 zygosities) regardless of diagnosis.

Small sample sizes of homozygous ε4 carriers are a common problem in APOE research due to the rare co-occurrence of two ε4 alleles [prevalence 2.9% (42)]. Only few studies exist that include sufficient sample sizes of ε4 homozygotes and further provide structural brain imaging data, and behavioral cognition measures. Despite the potential importance of MDD for APOE effects even fewer studies include mid-age subjects with MDD subsamples. Most studies including these aspects investigate elderly samples, while not differentiating ε4 zygosity (43, 44). To our knowledge only one study exists that included mid-age MDD subjects, however also not differentiating ε4 zygosity (41). We know of no study that has been published combining structural MRI data, and neuropsychological data in mid-age subjects, that includes homozygous ε4, and MDD subsamples.

The goal of the current work was to investigate the relationship of the APOE genotype with visuospatial working memory (vWM) and brain structural alterations first and foremost in the hippocampus in a young- to mid-age, non-demented sample. Further objectives were to identify potential moderators of these relationships, namely age and MDD diagnosis. Importantly homozygous ε4 carriers were operationalized as the genetic risk group and contrasted with a sample without ε4 allele as the literature outlined above suggests effects of the APOE genotype to be most pronounced for homozygotes. We hypothesized that 1) ε4 homozygotes show on average lower vWM performance compared to non-ε4 carriers, 2) vWM performance correlates positively with hippocampal volume and gray matter density, and 3) homozygous ε4 carriers exhibit smaller volume and gray matter density in the hippocampal area compared to non-ε4 carriers.

## Methods

### Participants

**Figure 1:**
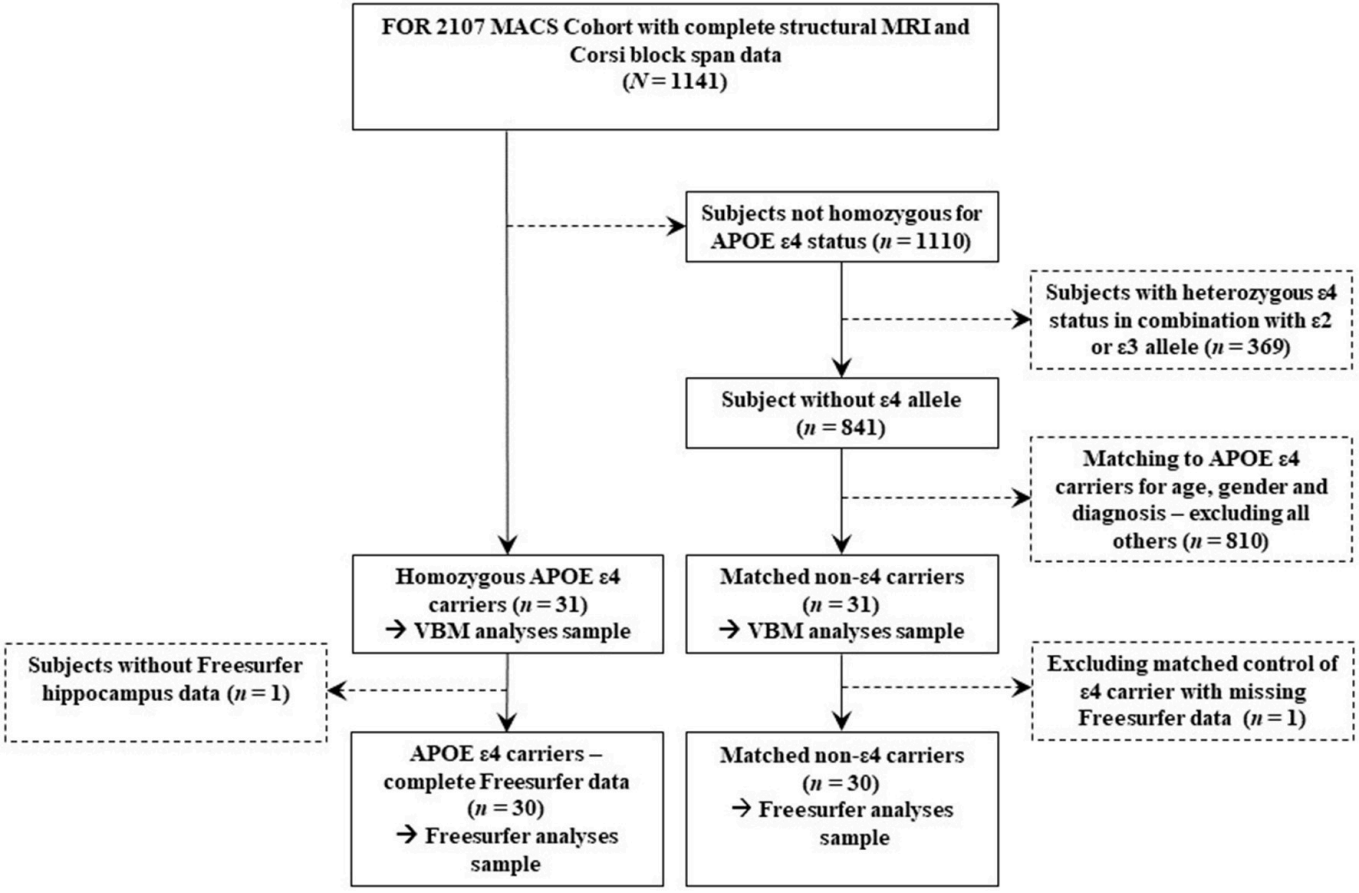
Sample flowchart showing all data exclusion steps for both genotype groups.

A sample of *N* = 1,141 adult subjects with complete cross-sectional MRI and neuropsychological data was available from the FOR2107 Marburg-Münster Affective Cohort Study (MACS) (45) which includes subjects with MDD and healthy control (HC) subjects. Exclusion criteria were any history of neurological (e.g., concussion, stroke, tumor, neuro-inflammatory diseases) and medical (e.g., cancer, chronic inflammatory or autoimmune diseases, heart diseases) conditions as well as substance-related addiction, and MRI contraindications. The study sample was selected based on the genotype of the APOE gene. Within the FOR2107 MACS cohort a study sample of *n* = 31 homozygous ε4 carriers was available which was contrasted with *n* = 31 non-ε4 carriers that were matched for age, sex, and diagnosis. For Freesurfer (FS) analyses a sample of *N* = 60 (*n* = 30 ε4 homozygotes; *n* = 30 matched controls) was available due to missing data for one of the ε4 homozygotes. The sample selection process is depicted in Figure 1.

The final sample of *N* = 62 included *n* = 24 MDD patients and *n* = 38 HC subjects. The mean age of the sample was 34.47 (*SD* = 13.48), ranging from 19 to 63 with 51.6% of the sample being female. A more detailed description of the distribution of demographic characteristics can be found in Table 1.

**Table 1:** Demographic characteristics of the final study sample.

	**ε4 homozygotes**		**Non-ε4 carriers**	
	***n***	**Mean age (*SD*)**	***n***	**Mean age (*SD*)**
**MDD**				
Male	7	41.86 (15.25)	7	41.71 (15.13)
Female	5	35.40 (18.12)	5	35.40 (18.12)
Total	12	39.17 (16.03)	12	39.08 (15.96)
**HC**				
Male	8	24.50 (2.78)	8	24.50 (2.78)
Female	11	36.64 (12.49)	11	36.64 (12.49)
Total	19	31.53 (11.30)	19	31.53 (11.30)

Subjects from the MACS cohort were recruited using advertisement in psychiatric hospitals, and outpatient therapeutic offices as well as advertisement in other public places and newspapers. Data were collected at the Departments of Psychiatry either at the University of Marburg or at the University of Münster. Before study participation, all subjects gave written and informed consent. All participants received a financial compensation.

### Materials and Procedure

#### Neuropsychological Assessment

The Corsi block tapping task (33) was administered in a forward and a backward version. This task is a well-established measure of vWM that has shown to be sensitive for cognitive decline associated with AD (46–48). A mean individual block span capacity was calculated over both version scores regarding a lack of evidence for conceptual differences between both version performances (49).

#### Clinical Assessment

A structured clinical interview for DSM-IV (SCID-I) was conducted with each participant in order to assess current and lifetime psychopathological diagnoses (50). All subjects allocated to the MDD group in this study either fulfilled the DSM-IV criteria for an acute major depressive episode or had a lifetime history of a major depressive episode. All HC subjects were ensured to be free from presence or any history of psychiatric disorders according to DSM-IV criteria.

#### Structural Image Acquisition and Processing

T1-weighted high-resolution anatomical images were acquired for all participants. Two MRI scanners were used for data acquisition located at the sites in Marburg and Münster with different hardware and software configurations. In Marburg, a 3T MRI scanner (Tim Trio, Siemens, Erlangen, Germany) was used combined with a 12-channel head matrix Rx-coil. In Münster, data were acquired at a 3T MRI scanner (Prisma, Siemens, Erlangen, Germany) using a 20-channel head matrix Rx-coil. Pulse sequence parameters were standardized across both sites to the extent permitted by each platform. Further details of the imaging procedures and implemented quality assurance protocol have been extensively described elsewhere (51).

Image preprocessing has been conducted using two complementary approaches that have frequently been described in our previous work: voxel-based morphometry (VBM) (52–54) using CAT12 (http://dbm.neuro.uni-jena.de/cat/) and FS (53, 55) based automatic segmentation (Version 5.3) with default parameters (https://surfer.nmr.mgh.harvard.edu/).

CAT12 preprocessing was done using default parameters: Briefly, images were bias-corrected, tissue classified, and normalized to MNI-space using linear (12-parameter affine) and non-linear transformations, within a unified model including high-dimensional DARTEL-normalization. The modulated gray matter images were smoothed with a Gaussian kernel of 8 mm FWHM. Absolute threshold masking with a threshold value of 0.1 was used for all second level analyses as recommended for VBM analyses (http://www.neuro.uni-jena.de/cat/). Image quality was assessed by visual inspection as well as by using the check for homogeneity function implemented in the CAT12 toolbox.

For all analyses using FS based segmentations, volumetric measures of the hippocampal region were used (56). Quality checks of segmentations were done visually and based on a statistical outlier analysis following a standardized protocol provided by the ENIGMA consortium (http://enigma.ini.usc.edu/protocols/imaging-protocols).

#### Genotyping and Quality Control

DNA was extracted from EDTA blood samples using the chemagic 360 instrument (PerkinElmer, Waltham, MA, USA). Individuals were genotyped using the Infinium PsychArray BeadChip (Illumina, San Diego, CA, USA) and standard protocols. Genotyping was performed at the Institute of Human Genetics, University Hospital Bonn, Germany. Clustering and initial quality control were conducted using GenomeStudio v.2011.1 (Illumina, San Diego, CA, USA) and the Genotyping Module v.1.9.4. Quality control procedures were implemented using PLINK (57) on the FOR2107 dataset. Samples with low genotype rates <95%, sex inconsistencies (X-chromosome heterozygosity), and genetically related individuals were not included in the present study. We also excluded SNPs that had a poor genotyping rate (<95%), strand ambiguity (A/T and C/G SNPs), a minor allele frequency (MAF) <1%, or that showed deviation from Hardy-Weinberg Equilibrium (*p* < 10^−6^). The quality controlled genotype data was imputed in the Michigan imputation server (https://imputationserver.sph.umich.edu) (58) using the Haplotype Reference Consortium reference panel (v3.20101123). The hard called genotypes for the single nucleotide polymorphisms (SNPs) rs7412 and rs429358 in APOE were extracted from the imputed dosage using PLINK. Both the SNPs were imputed with high accuracy (R^2^ > 0.99). Participants homozygous for the ε4 allele (ε4/ε4) were contrasted with subjects without an ε4 allele (genotypes ε2/ε2, ε2/ε3, or ε3/ε3).

### Statistical Analyses

#### APOE Status and Visuospatial Working Memory

In order to test our first hypothesis (expecting reduced vWM performance in ε4 homozygotes), APOE genotype was entered as a predicting factor for Corsi block span in a general linear model. Age, sex, and presence of MDD diagnosis were included as covariates in the model.

#### Visuospatial Working Memory and Hippocampus

The second hypothesis (positive correlation between vWM performance and hippocampus morphometry) was similarly tested using a general linear model. Total intracranial volume (TIV) and scanner site were used as covariates in addition to the covariates age, sex, and MDD diagnosis, while Corsi block span was used as the predictor variable. For this analysis step both (a) FS based analysis using hippocampal volumes as dependent variable (including hippocampal laterality as a within-subject factor in the model) and (b) VBM region of interest analysis of the hippocampus were conducted.

#### APOE Status and Hippocampus

Identical analyses as for the second hypothesis were used for the investigation of our third hypothesis (negative relationship between homozygous ε4 status and hippocampal morphometry), except that APOE status was used as a predictor instead of Corsi block span. Again the model was used for the prediction of (a) hippocampal volume, and (b) gray matter density (VBM) in two separate analyses in accordance with analyses testing the second hypothesis.

#### General Aspects and Exploratory Analyses

Generally, all analyses were based on a significance threshold of *p* < 0.05, unless noted differently. However, as all hypotheses were clearly one-sided, halved *p*-values are reported regarding the main hypotheses (effect of APOE status on vWM, effect of vWM on hippocampal morphometry, and effect of APOE status on hippocampal morphometry) if not specified otherwise. Any interaction terms, and whole brain effects tested for significance were regarded as exploratory and thereby tested two-sided.

VBM analyses were carried out using SPM12 (https://www.fil.ion.ucl.ac.uk/spm/). The hippocampal mask for VBM analyses was created by means of the WFU PickAtlas (59) according to the AAL-Atlas (60) and dilated 2 mm.

To control for multiple comparisons, threshold-free cluster enhancement (TFCE) corrections were applied for all VBM analyses using *N* = 1,000 permutations and a cluster size weighting of *E* = 0.5.

All further analyses were conducted with SPSS (version 25.0). Partial η^2^ values are reported as effect size measure for significant effects. Reported effect sizes of VBM analyses are based on extracted eigenvariates on cluster level.

## Results

### APOE Status and Visuospatial Working Memory

In order to test our first hypothesis vWM was predicted with APOE genotype in the above described model. Results yielded a significant main effect of APOE status [*F*_[1, 57]_ = 6.21, *p* = 0.02, η^2^ = 0.10] with lower Corsi block span in APOE ε4 carriers. Further, a main effect of diagnosis emerged [*F*_[1, 57]_ = 4.21, *p* = 0.05, η^2^ = 0.07] with lower mean block span in MDD subjects. The model yielded no further main effects (all other *p* > 0.53).

**Figure 2:**
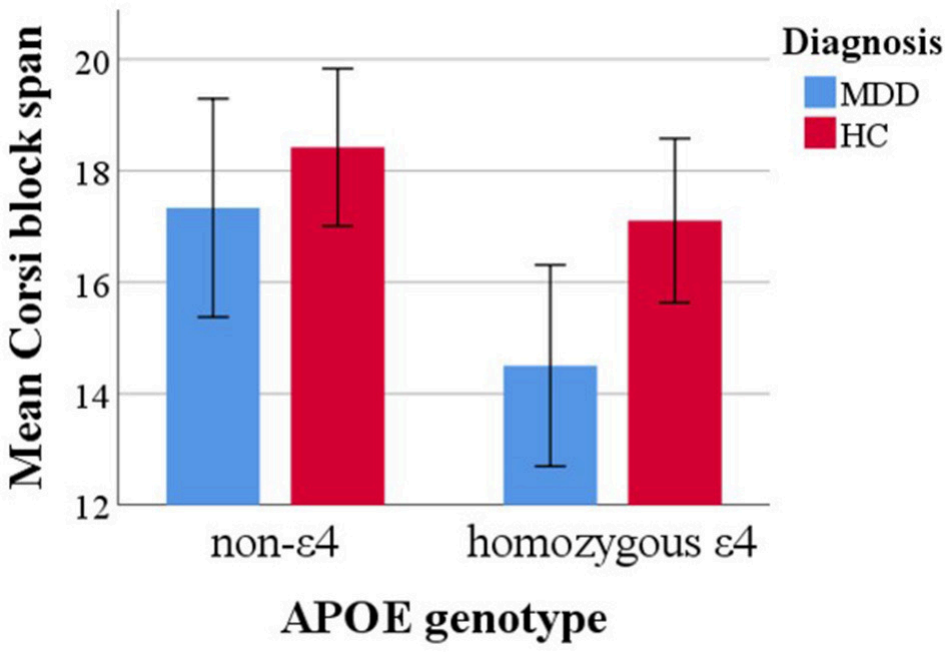
Mean Corsi block span capacity over APOE genotype and diagnosis groups. Error bars represent 95% confidence intervals.

When adding an interaction term between APOE status and age it turned out non-significant [*F*_[1, 56]_ = 0.21, *p* = 0.65], as was the interaction effect between APOE genotype and sex [*F*_[1, 56]_ = 2.22, *p* = 0.14]. Descriptively mean differences as a function of APOE status were more pronounced for MDD compared to HC subjects. However, testing an interaction term between APOE status and diagnosis also yielded a non-significant effect [*F*_[1, 5]_ = 0.93, *p* = 0.34]. Mean differences in Corsi span over APOE and diagnosis groups are depicted in Figure 2.

### Visuospatial Working Memory and Hippocampus

For our second hypothesis the general linear model described above was fitted with Corsi block span predicting hippocampal volume. Results yielded a non-significant effect of block span on FS derived hippocampal volume [*F*_[1, 53]_ = 0.34, *p* = 0.56]. The main effect of diagnosis was significant [*F*_[1, 53]_ = 6.63, *p* = 0.01, η^2^ = 0.11], with lower hippocampal volume in MDD compared to HC subjects. No significant effect of hippocampal laterality [*F*_[1, 53]_ = 0.01, *p* = 0.94], and no other significant main effect of interest was found (all other *p* > 0.47).

When interaction terms were added to the model, results yielded a non-significant interaction effects between Corsi block span and age [*F*_[1, 52]_ = 0.03, *p* = 0.86], and between Corsi block span and sex [*F*_[1, 52]_ = 0.69, *p* = 0.41]. Testing an interaction term between Corsi block span and MDD diagnosis also yielded a non-significant interaction effect [*F*_[1, 52]_ = 1.76, *p* = 0.19]. Exploratory analyses revealed that when removing TIV from the model, the main effect of Corsi block span on hippocampal volume becomes significant [*F*_[1, 52]_ = 3.49, *p* = 0.03, η^2^ = 0.06]. Further, when substituting hippocampal volume with TIV as the criterion variable the main effect of Corsi block span was significant [*F*_[1, 54]_ = 5.49, *p* = 0.02, η^2^ = 0.09], with higher Corsi block span being associated with higher TIV.

In order to further investigate our second hypothesis, model estimation was repeated within a VBM analysis. For the hypothesized main effect of vWM on hippocampal gray matter density the analysis yielded two marginally significant clusters just above a TFCE-corrected significance threshold of *p* < 0.05 (left: *k* = 132, *p* = 0.05, *x* = −38, *y* = −28, *z* = −15, TFCE = 203.05, η^2^_*cluster*_ = 0.18; right: *k* = 50, *p* = 0.07, *x* = 38, *y* = −24, *z* = −18, TFCE = 181.78, η^2^_*cluster*_ = 0.23).

**Figure 3:**
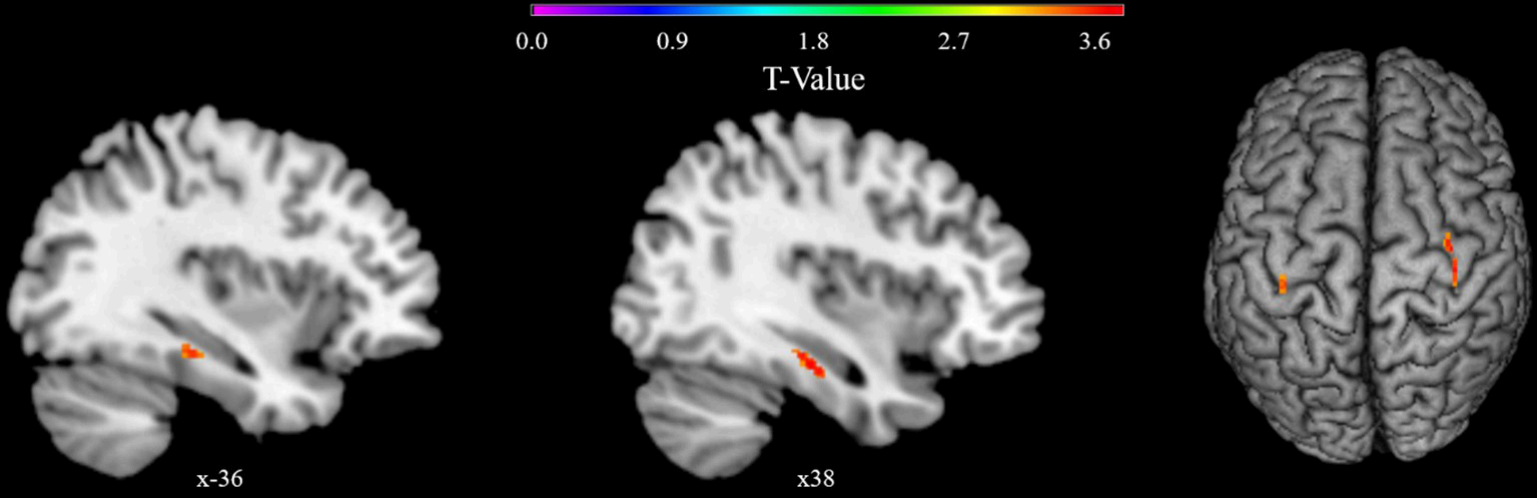
VBM whole brain results showing clusters with a positive correlation of gray matter density with Corsi span in the general linear model. Results are presented below an exploratory uncorrected significance threshold of *p* < 0.001 with a minimum cluster size of 20 voxels. Clusters are located in the left temporal inferior and fusiform gyrus and left hippocampus, and in the right parahippocampus, hippocampus, and fusiform gyrus.

Exploratory whole brain analysis for the main effect of vWM revealed three clusters below the exploratory uncorrected threshold of *p* < 0.001 with a minimum cluster size of 20 voxels (*k* = 31, *p* < 0.001, *x* = 36, *y* = −14, *z* = −28; *k* = 21, *p* < 0.001, *x* = 38, *y* = −22, *z* = −20; *k* = 20, *p* < 0.001, *x* = −38, *y* = −28, *z* = −15). Clusters were located in the right parahippocampus, fusiform gyrus, and hippocampus, as well as left temporal inferior and fusiform gyrus, and left hippocampus but not in any other region (see Figure 3).

### APOE Status and Hippocampus

No significant main effect of APOE status on hippocampal volume using the general linear model outlined above could be detected [*F*_[1, 53]_ = 0.67, *p* = 0.42]. However, a significant main effect of MDD diagnosis [*F*_[1, 53]_ = 7.87, *p* = 0.01, η^2^ = 0.13] was found, with smaller hippocampal volume in the MDD group compared to the HC group. No within-subject effect of hippocampus laterality [*F*_[1, 53]_ = 0.33, *p* = 0.57], and no other main effects of interest emerged (all other *p* > 0.41).

**Figure 4:**
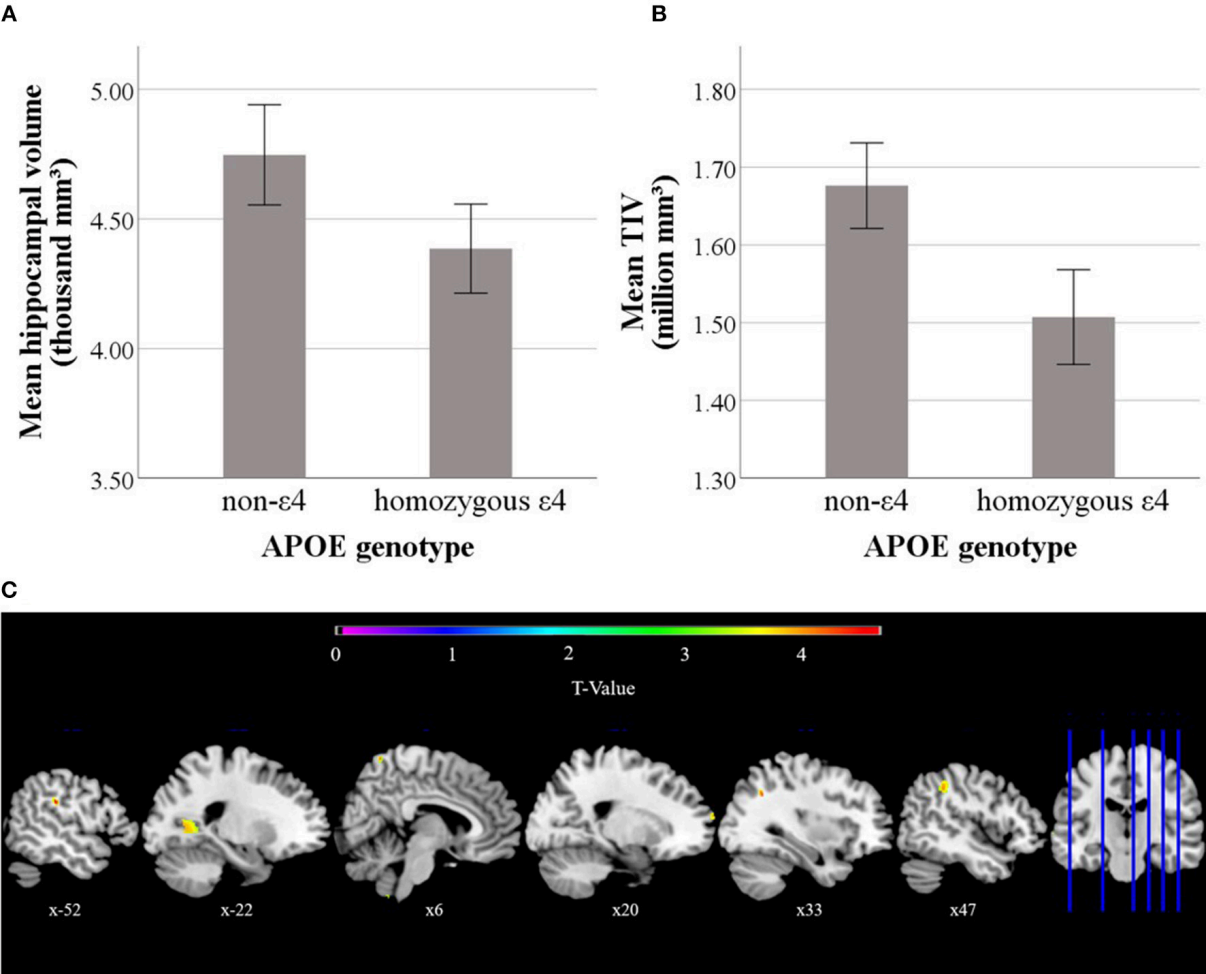
Mean hippocampal volume **(A)**, and mean total intracranial volume (TIV) volume **(B)** differences over APOE genotypes. Depicted hippocampal volumes were calculated as mean volumes between left and right laterality. Error bars represent 95% confidence intervals. VBM results of the contrast that ε4 homozygotes have lower gray matter density than non-ε4 carriers are depicted at the bottom **(C)**. Presented clusters correspond to the clusters described in Table 2.

When adding an interaction term between APOE status and age to the model this yielded a non-significant interaction effect [*F*_[1, 52]_ = 0.12, *p* = 0.73]. Adding an interaction term between APOE status and sex also yielded a non-significant effect [*F*_[1, 52]_ = 0.03, *p* = 0.86]. The interaction between APOE status and diagnosis was also non-significant [*F*_[1, 53]_ = 0.05, *p* = 0.82]. Exploratory analyses revealed that when removing TIV from the model, hippocampal volume was significantly predicted by APOE genotype [*F*_[1, 54]_ = 11.64, *p* = 0.001, η^2^ = 0.18]. In order to further explore the role of TIV, the same model as described above was applied to predict TIV instead of hippocampal volume in a subsequent analysis. This yielded a significant main effect of APOE status [*F*_[1, 54]_ = 25.59, *p* < 0.001, η^2^ = 0.32], with lower mean TIV in ε4 homozygotes. Mean TIV and hippocampal volume over APOE genotype is depicted in Figure 4.

VBM model estimation yielded no clusters beyond significance threshold for the APOE genotype contrast at a TFCE-corrected significance level of *p* < 0.05. Exploratory whole brain VBM analysis using the same model yielded eight clusters beyond an exploratory uncorrected threshold of *p* < 0.001 and a minimum cluster size of 20 voxels (see Table 2). No clusters remained significant when applying TFCE corrections on whole brain level. When using the exploratory uncorrected threshold at *p* < 0.001 with a minimum cluster size of 20 voxels no clusters emerged for the inverted contrast of greater gray matter density in the ε4 homozygotes on whole brain level.

**Table 2:** Clusters above exploratory uncorrected significance threshold *p* < 0.001 with minimum voxel number of 20 for the contrast of lower gray matter density in homozygous APOE ε4 carriers.

**Main area**	***k*_**E**_**	**Peak voxel coordinates**			**Peak voxel coefficients**	
		***x***	***y***	***z***	***t***	***p***
Supramarginal gyrus	51	−52	−30	30	4.62	0.000
Angular gyrus	26	33	−57	34	4.24	0.000
Supramarginal gyrus	119	48	−40	42	3.74	0.000
Precuneus	20	6	−58	68	3.70	0.000
Superior frontal gyrus	31	20	69	15	3.66	0.000
Calcarine	311	−24	−62	8	3.64	0.000
Cerebellum	46	2	−50	−58	3.40	0.000
Middle temporal gyrus	26	−63	−22	0	3.24	0.001

*Uncorrected p-values of peak voxel differences are reported*.

## Discussion

The main finding of the present study is that APOE ε4 homozygosity is associated with lower vWM ability already in young to mid-age, non-demented subjects. Effect size estimates indicate a medium sized effect. This effect seems to be age-independent within the age range of our sample (19–63 years) and is of comparable magnitude in the MDD subsample as in the HC sample. Similarly, we found lower vWM capacity in people with MDD diagnosis compared to HC subjects. Although some evidence was found that hippocampal gray matter density was associated with vWM, no specific relationship was detected between vWM and hippocampal volume when including TIV as a covariate. Furthermore, both measures of hippocampal structure (gray matter density and volume) did not significantly differ between genotypes in our sample when controlling for global brain structural measures. In addition, a specific effect of MDD diagnosis on hippocampal volume was found which appeared to be independent from APOE genotype.

Previous work investigating the influence of APOE genotype on cognitive abilities present a general trend of lower abilities in various cognitive domains for ε4 carriers (18, 20, 21, 24, 41, 61). Although some studies also find differences regarding the visuospatial domain (24, 25) the evidence is more inconsistent in this field and a meta-analysis even concluded that there is little evidence for an impact on the visuospatial domain by the APOE gene (21). This may hold true for heterozygous ε4 carriers compared to non-ε4 carriers (as most studies compare these groups). However, little research exists including valid sample sizes of ε4 homozygotes. The current study provides a comparatively large sample size of ε4 homozygotes. It revealed medium sized group differences for the Corsi block-tapping task performance. As measures of visuospatial ability vary between studies it cannot be ruled out that the effect shows more in the Corsi task than other tasks of visuospatial performance that might tax domain components differently (for example more or less taxing of executive components of working memory).

The findings of the current study suggest that both APOE status and MDD diagnosis have deteriorating effects on vWM, which appear to be of an additive nature. In contrast a longitudinal study investigating the interplay between depression and APOE status on AD risk, proposes an interactional relationship, as that depression constitutes a risk factor only in ε4 carriers (39). Regarding cross-sectional effects in mid-life, our results are in line with other evidence proposing that MDD and ε4 effects on cognitive ability are rather additive (41, 62) suggesting that interactional effects might only show later in life and/or in combination with demential processes.

The literature addressing interaction effects between APOE genotype and age on cognitive ability is very heterogeneous depending on cognitive measures and age span under investigation. Most studies finding cognitive decline effects in APOE ε4 carriers have investigated elderly samples from 60 years upwards (63). Evidence is mixed for mid-age samples (61) while a meta-analysis regarding children and young adults suggest no cognitive differences dependent on APOE status (64). For the sample investigated in the current study no moderating influence of age on APOE effects on cognition became evident. Genotype effects were further not found to be moderated by sex.

Regarding the relationship between vWM and brain structural measures the results found here hold several important insights. Two measures of hippocampal morphometry were used in the current study and findings varied depending on the measure. While hippocampal volume was not associated with vWM capacity, gray matter density was marginally significantly increased in higher vWM capacity individuals. Furthermore, exploratory additional analyses indicated that the effect on hippocampal volume was masked by the covariate TIV (possibly suggesting a more unspecific, global association), while the exploratory whole brain VBM analysis suggested a specific effect located in the hippocampal and parahippocampal area (as no other areas were associated with vWM capacity). Findings suggest a global correlate of vWM regarding volumetric measures, while gray matter density seems to be associated with vWM specifically in the hippocampal and parahippocampal regions. Other findings suggesting a local link between the hippocampus and vWM capacity have used different measures of brain function (9, 34) and structure, as lesions (10, 35), gray matter density (9), and volume (36).

The pattern of results found in the current study suggests that although the ε4 allele may be associated with lower vWM capacity, the neuronal locus of this effect is not the hippocampal region specifically. Previous findings suggest a robust deteriorating APOE ε4 effect on hippocampal structure that supposedly is most pronounced for ε4 homozygotes (30, 31). However, in the current study we were unable to replicate group differences of hippocampal morphometry in dependence of APOE status (neither for a volumetric, nor for a gray matter density measure) after controlling for global brain measures. This finding is particularly surprising since we contrasted ε4 homozygotes with non-ε4 carriers. One possible explanation is given by Cherbuin et al. (30): the authors reviewed studies showing an effect and studies with contradicting findings, and propose that a potential effect of APOE genotype on hippocampal alterations may show only in a narrow age range around ~70 years. It is suggested that samples below the age of 60 years may not be affected yet, while an APOE effect may be masked by more general age-related alterations in samples over 80 years (30). This interpretation would be in line with our findings as most participants of our sample are considerably younger. A different explanatory approach arises from our exploratory analyses that revealed that the relationship between APOE status and hippocampus is covered by the covariate TIV that may have too much explanatory variance in common with the hippocampal volume. One interpretation of this finding is that the APOE genotype affects the cerebral morphometry on a more global level. This interpretation is in line with a brain reserve hypothesis that implies that a globally increased brain volume serves as a protective factor regarding demential processes (65). Although this hypothesis has been discussed controversially (66, 67) our findings suggest that a protective effect of the APOE genotype (not having an ε4 allele) regarding AD might be mediated by global TIV. Further, it is also possible that the smaller TIV of ε4 homozygotes does not constitute a causal link between genotype and AD risk but is a byproduct of other neuronal processes. Either way, although the rational of controlling for TIV in brain morphology analyses seems straight forward, one should be aware that controlling for TIV variance might also covert morphometric effects of interest (68).

Causality of a genotype on an associated present phenotype cannot be definitely concluded as linkage disequilibrium with other causal factors cannot be ruled out (69). However, there is some evidence for a causal effect of the APOE genotype on visuospatial ability coming from animal models showing a reduced performance in a water maze task in APOE knockout mice with synthetic human APOE ε4 compared to APOE ε3 (70). Even though the translation to humans is not certain this finding supports the notion of a causal effect of APOE genotype on cognition in the current study. Owing to the cross-sectional nature of our data the reported link between brain structure and vWM cannot be interpreted regarding to its causal direction.

Major strengths of the current work comprise the relatively large sample of ε4 homozygotes compared to previous studies (bearing in mind that this cohort was obtained from a total of *N* = 1,141). Another major strength lies in the inclusion of cognitive as well as brain structural data.

The results of the current study underlie several limitations which have been addressed in part. Firstly, only homozygous ε4 carriers were compared to non-ε4 carriers. Consequently, no conclusions can be derived regarding a potential dose effect of the ε4 allele status, and also no differential conclusions can be made about subgroups of different non-ε4 genotypes. Further, despite the availability of a large initial sample only a relatively small study sample of ε4 homozygotes could be included due to a naturally low occurrence rate of the ε4 allele. Thus, small effects are more difficult to detect and it cannot be ruled out that associations with small effect sizes can be found in higher-powered studies that were not significant in the current study. This could for example concern a potential interaction effect of MDD diagnosis and APOE status that was descriptively looming in the current study but did not become significant. A potential interaction of age and APOE status could particularly be affected by small sample sizes because samples have to be sufficiently powered across the whole age-range under investigation. The current study does not allow conclusions about age effects below the age of 19 and above the age of 63. Further, interactions of small effect sizes may be present within this range.

Furthermore, conclusions derived regarding the vWM capacity are to be interpreted with caution as the concept of vWM is rather heterogeneous and results may vary over different measures of vWM.

## Conclusions

Combined results suggest that a detrimental effect of APOE genotype on vWM capacity exists and might be mediated by global volumetric measures rather than by region-specific volumetric or gray matter density measures. More specific hippocampus alterations as a function of APOE status may occur later in life but do not become evident in our young- to mid-age sample.

More longitudinal high-powered studies investigating the interplay between the APOE genotype, MDD and cognitive deficits, as well as neuronal processes involved are needed. It is important that future studies include sufficient sample sizes of ε4 homozygotes. Large multi-centered studies constitute a promising approach for achieving this goal.

## Ethics Statement

This study was carried out in accordance with the recommendations of the Medical Faculty of the University Münster with written informed consent from all subjects. All subjects gave written informed consent in accordance with the Declaration of Helsinki. The protocol was approved by the Medical Faculty of the University Münster.

## Author Contributions

JG, RR, NO, UD, BB, TK, MN, MR, IN, AK, and AJ: study concept and design. JG, NO, RR, UD, KD, KF, DZ, SM, VE, ES, LF, KB, DG, AT, SS, FS, TM, DY, and EF: acquisition, analysis, or interpretation of data. JG, NO, and UD: drafting of the manuscript. RR, TK, AJ, BB, AF, VE, SW, AK, TH, KF, JR, DZ, KD, CK, DG, SM, ES, LF, TH, AK, IN, SM, FS, TM, DY, EF and MR: critical revision of the manuscript for important intellectual content. JG, NO, RR, and UD: statistical analysis. UD, NO, RR, and TK: obtained funding. RR, KF, KD, DZ, SM, VE, ES, LF, KB, JG, CK, HK, DG, TH, AT, SS, FS, TM, DY and EF: administrative, technical, or material support. UD, NO, BB, TK, MN, MR, IN, AK, and AJ: study supervision. All authors read and approved the submitted version of the manuscript.

### Conflict of Interest Statement

The authors declare that the research was conducted in the absence of any commercial or financial relationships that could be construed as a potential conflict of interest.
